# (No) Empathy for the Monkey? Book Review of “The Cultural Origins of Human Cognition”

**DOI:** 10.5964/ejop.v11i2.990

**Published:** 2015-05-29

**Authors:** Constance de Saint-Laurent

**Affiliations:** a University of Neuchâtel, Neuchâtel, Switzerland

[Other p1]About ninety years ago, Lev Vygotsky stated that most of our higher mental functions are culturally derived, and therefore that the uniqueness of humanity was in our ability to create history (Vygotsky, 1934/1997). Although he was more interested by how phylogeny and culture merge during ontogeny, he nevertheless left us with one important question: what happened in the evolution of the human race that allowed the development of culture, and our inscription into history? In 1999, Michael Tomasello presented in his book ‘The Cultural Origins of Human Cognition’ more than ten years of research he did on the subject, comparing young children’s cognition and the abilities of great apes. He came to the conclusion that the only significant cognitive difference is our capacity to share and understand intentions – or at least our willingness to do so (Tomasello, Carpenter, Call, Behne, & Moll, 2005), a capacity that is essential for the development of *cumulative cultural evolution*^i^. However, it will be argued here that Tomasello overlooked important findings in the mechanisms of evolution as well as in the cognition and socialisation of great apes, making his theory stand on arguable foundations. By understating the social skills of other hominids and simplifying the relation between culture and theory of mind, the importance of other cognitive abilities in this debate is often forgotten.

Although Tomasello’s book has now been published some 16 years ago, this review was motivated by the recrudescence of social and cultural arguments based on the comparison between humans and great apes. From education (e.g., Bruner, 1996) to sociology (Latour, 2007), no field of the human and social sciences seems to escape the need to compare us with our closest relatives, concluding that we have greater social and empathic skills. However, recent research has shown that such results are often biased, using definitions of society and culture tailored to exclude non-humans (King, 2002) and ignoring the fact that experimental contexts may not tell much about great apes’ abilities in the wild. Because of the importance of Tomasello’s book on psychologists’ discourses about great ape cognition, its review is still relevant today. However, to do justice to the author’s body of work, recent alterations he made to his theories in the meantime have also been taken into account.

Tomasello’s experiments on nonhuman primates and young children led him to the conclusion that if all hominids can understand the other as an intentional agent, only humans can share intentionality (Tomasello et al., 2005). If great apes have, in theory, the ability to share intentionality, they do not show any interest in doing so, and tend to present an understanding of others’ intentions only when rewarded with food. The ability to ‘co-act’, through the coordination of two agents’ goals, allows not only for the apparition of culture, but also for cumulative cultural evolution. Indeed, it appears that a few animal species can produce culture, even though it largely depends on how we define it (Boyd & Richerson, 1996), but only humans can produce what Tomasello calls the “ratchet effect”: individuals not only produce new behaviours, but these behaviours are maintained by the group to be later improved by another individual. It is this unique spiral process that allows us to ‘stand on the shoulders of giants’ and develop knowledge that could not be produced by a single individual.

Tomasello’s starting point is that evolution, on a short period of time that is two million years, could only produce one main difference – that his book aims to find – between Homo sapiens and the other hominids, especially if we consider that we share around 99% of our DNA with Bonobos, our closest relatives. However, three objections can already be made. First, evolution can be accelerated by processes such as niche construction (Odling-Smee, Laland, & Feldman, 2003) or group selection, undermining Tomasello’s timescale argument. Second, sharing almost all our genome with other primates is not as meaningful as it appears: we share, for instance, 50% of our genomes with bananas… Indeed, what matters when considering genotypes are the genes responsible for global organisation (Marks, 2003), which means that a minimal genotypic difference can lead to the expression of completely different phenotypes. Moreover, the exclusive use of statistical inferences in genetics limits the conclusiveness of the obtained results (Kupiec & Sonigo, 2000). Third, the strongest competition in evolution is between close species, as they fill the same place in nature (Darwin, 1859/2004). Other hominans do not seem to have been especially inscribed in cultural evolution (Foley & Lahr, 1997), although showing signs of a theory of mind (e.g., burial of the dead, painting, etc.) Therefore, if we are to share all of our characteristics but one with other hominids, it is more likely to be with other hominoids that are now extinct.

In order to determine this unique difference between humans and other primates, Tomasello compares how children and great apes react to different cognitive tasks involving socialisation and intentionality. That is, apes and children are faced with a seemingly incompetent experimenter and are required, to “successfully” pass the experiment, to read the intentions of the researcher and to help him in his (rather simple) task. And, here, the conclusion is clear: if children start extremely early on – around 6 months – to enthusiastically help the experimenter, apes do so only when rewarded with food. Thus, humans have a predisposition towards socialisation that allows them to share intentionality. Why, then, can apes in captivity and under certain other conditions understand intentions and yet are not very willing to do so? The effects of enculturation are often pointed to in this case, and it has been argued either that treating apes as intentional agents may lead them to see others as being so as well (Tomasello & Call, 1996, 2004), or that it provokes a simple behavioural change (Bering, 2004). Nevertheless, two other possibilities have been overlooked.

First, displaying the same ability does not necessarily mean that it is possible through the same processes and it is indispensable to look at how the ability evolves during ontogeny (Vygotsky & Luria, 1934/1994). However, if much data is available for the early stages of development, it is increasingly difficult to separate ontogenesis and sociogenesis when getting to later stages (Featherman & Lerner, 1985), and no study includes longitudinal data on the socialisation or enculturation of great apes. Therefore considering, as Tomasello does, that we understand the intentions of others through the same process as apes, but that we only show more interest in doing so, seems to overlook the fact that drawing conclusions from behavioural observations must be carefully done, and that further data is necessary. Second, recent experiments done in natural settings – that is, in the wild – and experiments with enculturated apes have shown a quite different picture of great apes’ socialisation. They can evaluate the knowledge of others (Crockford, Wittig, Mundry, & Zuberbühler, 2012), intentionally transmit information to them (Schel, Townsend, Machanda, Zuberbühler, & Slocombe, 2013), integrate contextual elements to understand others (Arnold & Zuberbühler, 2013), show empathy (Pruetz, 2011), for instance. What motivates them, in the wild, to adopt collaborative behaviours seems to be friendship and kindship (Schel et al., 2013). Is it possible, then, that great apes simply do not want to collaborate with the experimenter yet would be able to do so?

Tomasello’s final assumption is that the necessary and only condition for the apparition of cultural evolution is the emergence of theory of mind, as they both depend on socialisation. This theory, with little modifications, is widely shared in psychology (e.g., Mead, 1930/1977 and Freud, 1915/1968 to quote only a few key thinkers) although a careful study of the socialisation and cognition of great apes shows the necessity of some alterations. It is certainly not our aim here to deny the social origins of the theory of mind or cultural evolution but to illustrate that the relation that binds them is not as simple as presented in Tomasello's work and that the importance of other cognitive abilities should not be that easily disregarded. It has been clearly shown that apes dispose of highly developed social skills, which involve understanding the intentions of others (Tomasello et al., 2005), the use of signs (King, 2002), and resilience in the socialisation process (Burks, Bloomsmith, Forthman, & Maple, 2001). They also show a positive reaction to the mirror test (Patterson & Cohn, 1994), and display empathy (Patterson & Gordon, 1993). If this does not mean that apes are self-aware in the way humans are, or that their social skills match ours, it suggests at least that apes might have access to a primitive theory of mind. And this without being able to develop any kind of cultural evolution, which cast a serious doubt on how straightforward the relation between self-awareness and other uniquely human abilities really is. One suggestion has been that although great apes have culture, they are not aware that they do: what they are missing, then, is a metalevel of understanding of their cultural ability (Gruber, Zuberbühler, Clément, & van Schaik, 2015).

This points towards an alternative explanation of our differences with great apes. Indeed, if most of the experiments on great apes tend to demonstrate how skillful they are, little attention is given to how long it takes the experimenters to make them achieve a task (see Schroeder's (1978) documentary, for instance, for some footage of Patterson's work with gorillas). It has often been considered as an effect of apes' 'laziness', and their little interest in non-rewarded exercises. However, when put in relation with what has been presented here, it seems that the importance of purely cognitive abilities has been overlooked. It is not only necessary to have a theory of mind or to be enculturated to point objects to others, but also to be able to be intentionally attentive. In order to speak a language or develop a 'generalised other', one needs to understand concepts. Finally, understanding intentions cannot be done without a context, which requires a plethora of cognitive abilities to apprehend the cultural interpretation of the 'here and now'. But, already when Lev Vygotsky was demonstrating in 1934 that most of our capacities are culturally developed, he insisted upon the fact that unspecific abilities, such as formation of concepts or logical memory, had been selected during phylogeny and it was these which were the building blacks of our cultural evolution.

To conclude, Tomasello’s work provides extremely interesting data and stimulating hypotheses. However, although his book is well argued and raises some valuable points, other important findings and theories that have been overlooked cast a shadow on a key part of his argument. It cannot be denied that socialisation is a cornerstone of the evolution of our species. Comparing how eager children brought up in isolation are to communicate (Cadland, 1993)^ii^ with the aggressiveness of apes in the same situation (Burks et al., 2001) leaves little doubt on this point. And there seems to be a little jump from here to the very comforting conclusion that, maybe, what makes humankind stand out from animal kind are our social and empathic skills. However, existing findings do not allow us to rule out just yet that we may be, after all, just very clever apes.
